# Genetic Diversity of Eight Domestic Goat Populations Raised in Turkey

**DOI:** 10.1155/2016/2830394

**Published:** 2016-03-22

**Authors:** Zafer Bulut, Ercan Kurar, Yusuf Ozsensoy, Vahdettin Altunok, Mehmet Nizamlioglu

**Affiliations:** ^1^Faculty of Veterinary Medicine, Department of Biochemistry, Selcuk University, 42031 Konya, Turkey; ^2^Meram Faculty of Medicine, Department of Medical Biology, Necmettin Erbakan University, 42080 Konya, Turkey; ^3^Faculty of Veterinary Medicine, Department of Biometrics and Genetics, Cumhuriyet University, 58140 Sivas, Turkey

## Abstract

The objective of this study was to determine the intra- and intergenetic diversities of eight different goat populations in Turkey including Hair, Angora, Kilis, Yayladag, Shami, Honamli, Saanen, and Alpine. A total of 244 DNA samples were genotyped using 11 microsatellites loci. The genetic differentiation between breeds was considerable as a result of the statistically significant (*P* < 0.001) pairwise *F*_ST_ values of each pair of breeds. Exceptionally, *F*_ST_ values calculated for Honamli and Hair breeds were statistically nonsignificant (*P* > 0.05). Heterozygosity values ranged between 0.62 and 0.73. According to the structure and assignment test, Angora and Yayladag goats were assigned to the breed they belong to, while other breeds were assigned to two or more different groups. Because this study for the first time presented genetic data on the Yayladag goat, results of structure analysis and assigned test suggest that further analyses are needed using additional and different molecular markers.

## 1. Introduction

While goat breeding has decreased between 1991 and 2009, the number of Hair goats continuously and significantly increased between 2009 and 2014 in Turkey. Among goat populations, Hair goat is the predominant (98%) type raised in Turkey [1]. Although Hair goat is most widely reared in the Mediterranean, Aegean, and Southeastern Anatolian regions, it is considered the most common native breed raised nationwide. Angora goat is raised in the Central Anatolia region, primarily in Ankara, as well as in a few provinces of the Southeastern Anatolia and Eastern Anatolia regions [2]. Kilis goat is distributed in the Southeastern Anatolia region, primarily in Gaziantep, Kilis, and Hatay provinces. Honamli goat is reared in the Mediterranean region, in the foothills of the Taurus Mountains, primarily in Konya, Isparta, and Antalya provinces [2]. Compared to other populations, Saanen, Alpine, Shami, and Yayladag goats are rarer in Turkey; however, other goat populations, except for Hair and Tiftik goats, have not been included in TUIK data [1]. In Turkey, there were no data or information particularly about the population reported as Yayladag goat in the present study. Yayladag and Shami goats are raised in the southern part of Turkey, mainly in Hatay Province and close to the Syrian border. Phenotypic, population, and geographical properties of these breeds were described in detail elsewhere [3, 4].

The earliest phylogenetic analysis of goat populations in Turkey comprises protein and enzyme polymorphism [5–7]. However, microsatellite markers are widely used in genetic characterization studies. The genetic relation between different goat populations was investigated using microsatellite markers in various countries [8–11]. It was reported that microsatellites had been most frequently used in genetic characterization studies conducted in Asian and African countries [12]. In Turkey, genetic diversity studies with microsatellites had been conducted in goat [13], cattle [14, 15], and horse [16] populations.

Determination of genetic structure and genetic characterization of animals is the first step in developing gene sources protection strategies. Therefore, the present study aimed to investigate genetic diversity among some goat populations in Turkey using microsatellite markers. Also, this study is the first report regarding Yayladag goat population.

## 2. Materials and Methods

### 2.1. Samples

In the present study, a total of 244 blood samples, which were obtained from Kilis (*n* = 32), Yayladag (*n* = 32), Shami (*n* = 32), Honamli (*n* = 32), Saanen (*n* = 28), Hair (*n* = 32), Angora (*n* = 43), and Alpine (*n* = 13) goat populations, were drawn into tubes containing K_3_-EDTA. DNA isolation was performed using standard phenol/chloroform method [17]. The study was approved by Selcuk University, Veterinary Faculty, Experimental Animal Research Ethical Committee (decision number: 2015/69).

### 2.2. Methods

Genomic DNAs were amplified by Polymerase Chain Reaction (PCR) using 11 microsatellite markers (Table 1), which were selected from the list recommended by the United Nations Food and Agriculture Organization (FAO) and International Society of Animal Genetics (ISAG). As per the PCR protocol, 1x Mg^++^ free PCR buffer (Fermentas), 200 *μ*M dNTP (Fermentas), 1.5 mM MgCl^++^, 0.375 unit* Taq* polymerase (Fermentas), 5 pmol of each primary pair (Table 1), and 50–100 ng template DNA were used in a single reaction. Each PCR reaction was prepared as 15 *μ*L in volume.

PCRs were performed at two steps using MJ Research PTC-200 Thermal Cycler. After a complete denaturation at 95°C for 2 minutes, step I consisted of denaturation at 94°C for 45 seconds for five cycles, annealing at 59°C for 45 seconds, and elongation at 72°C for 30 seconds. Step II consisted of a total of 30 cycles, each including 94°C for 30 seconds, 60°C for 30 seconds, and 72°C for 20 seconds. Finally, a complete adenylation was enabled by keeping the samples at 72°C for 10 minutes.

The resulting PCR products were loaded onto a Beckman Coulter CEQ–8000 Genetic Analysis System and allele genotypes were identified eluting by capillary electrophoresis.

### 2.3. Statistical Analysis

Number of alleles (Na), expected (He) and observed (Ho) heterozygosity levels, *F*-statistics, factorial correspondence analysis (FCA), phylogenetic trees (NJT), and structure analysis were determined using GenAlEx6 [18], Population 1.0 [19], TreeWiev [20], GENETIX 4.0 [21], and Structure v2.2 [22] package programs.

## 3. Results

**Figure 1:**
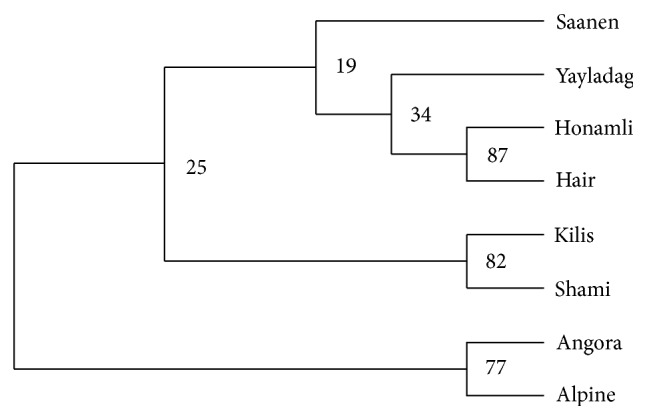
Neighbor-joining tree indicating phylogenetic relationship between Turkish native goat breeds.

**Figure 2:**
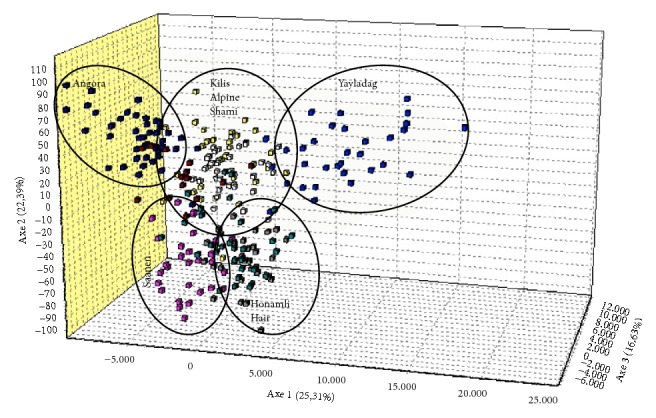
The FCA plot of the populations.

**Figure 3:**
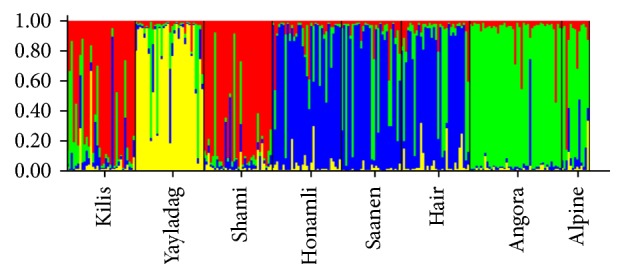
Structure analysis.

Among general population parameters, number of observed alleles (Na; Table 2), expected (He) and observed (Ho) heterozygosity levels (Table 3), NJT (Figure 1), FCA (Figure 2), and structure (Figure 3) were summarized.

In the present study, a total of 160 different alleles were observed and the mean Na was found to be 14.55. The highest number of alleles (33 alleles) was observed in BM6444, whereas the lowest number of alleles (10 alleles) was observed in McM527 and ILSTS087 markers.

It was determined that the mean Ho level changed between 0.188 and 0.969, whereas the mean He levels were observed between 0.427 and 0.917 (Table 3). Mean Ho and He levels were similar in general and heterozygosity among populations was generally high.

In order to evaluate genetic variation between populations, Wright's *F*-statistics was used and *F*_ST_ values were between 0.005 and 0.125 (Table 4). Total *F*_ST_ value (0.075) calculated for all loci was found to be statistically important (*P* < 0.001).

When the tree showing phylogenetic relation was analyzed by neighbor-joining tree (NJT) method using *D*_*A*_ genetic distance values of Nei, it was observed that the populations have been clustered in three main groups (Figure 1). Among these, Saanen, Yayladag, Honamli, and Hair were clustered together. The other groups including Kilis and Shami, Angora and Alpine populations were separated in different radiation.

Factorial correspondence analysis (FCA) was performed to determine the genetic relation between the individuals used in the study and each individual was placed on 3-dimensional plane according to their genotypes (Figure 2). Individuals of Ankara, Saanen, and Yayladag populations usually formed their own groups. However, it was observed that the subjects of other goat populations were grouped close to each other but did not much differ.

Structure test was performed to identify which population or populations the study subjects belong to as well as group them (Figure 3). *K* = 4 analyses revealed that all populations differed from each other and that Angora, Alpin, and Yayladag populations in particular are more pure breed compared to the others.

## 4. Discussion

The mean number of alleles observed in the study (14.55) and the number of alleles at each loci (10–33) were close to the findings of the other study, which was also conducted in Turkish goat populations using 20 microsatellite markers [13]. Three loci (CSRD247, MAF70, and INRA023) were the same between these studies and numbers of alleles were similar, although the highest number of alleles was reported between 7 and 24 in other goat populations [8, 23–28].

The mean Ho and He values (0.690 and 0.733, resp.) found in the present study were higher than those found in goat genetic characterization studies conducted in different countries using different numbers of microsatellites [29–31]. Moreover Na, Ho, and He values reported in almost all Asian and African countries [12] were lower than those obtained in the present study. These results indicate higher genetic diversity among Turkish goat populations. The reason for this might be the facts that Turkey is located on migration route and is close to the initial domestication centers [32, 33].

In a genetic study conducted using 13 microsatellites, 6 indigenous Iranian goat populations were separated into two main groups based on phylogenetic tree and FCA analysis [34]. Mitochondrial DNA analysis of local Chinese goat populations [35] revealed that they formed 4 haplogroups and the results were consistent with archeological and genetic studies. In addition, genetic characterization studies, which were performed using 30 microsatellites [36] reported that the populations, which were raised in northwest and southwest as two sets in accordance with their geographical regions, heaped up within themselves.

In the present study, it was observed that Honamli and Hair goat populations grouped together on the NJT. This suggests that there is high relationship between Honamli and Hair goats and that gene flow is possible as they are raised in close geographical regions. In addition, FCA graph demonstrated that Honamli and Hair goats grouped together in line with these findings. Assignment test was performed to identify the populations using genotypic data and it was observed that animals were generally (93%) assigned in their own populations. It was determined that 8 different populations, which were used also in the structure test, were discriminated from each other and that Angora, Alpine, and Yayladag were more pure breed compared to other populations. Results of assignment and structure analyses revealed that microsatellites used in the study are very useful in discriminating the populations.

There is no data or information in Turkey about Yayladag goat population used in the present study. However, previous research [37, 38] indicated that Hatay goat was obtained by hybridization of Hair × Kilis. However, Yayladag does not seem to be genetically so close to Hair or Kilis. Although they appear in the same group on NJT, Yayladag is grouped as a different population in the FCA and structure analyses. It was thought that suitable geographical structure of Hatay province for goat breeding and presence of many populations may be effective in the formation of a new genotype.

In conclusion, microsatellites are quite reliable markers to be used in the studies to investigate genetic variety and genetic structures of populations and to determine whether the subjects belong to the claimed populations. In addition, there is need for further studies using different markers system to obtain additional data on Yayladag goat.

## Figures and Tables

**Table 1:** Microsatellites used in the study.

Locus	Chromosome	Primer sequence		Allele range (bp)
		Forward (5′ → 3′)	Reverse (5′ → 3′)	
MAF70	4	cacggagtcacaaagagtcagacc	gcaggactctacggggcctttgc	134–168
INRA023	3	gagtagagctacaagataaacttc	taactacagggtgttagatgaact	196–215
SPS113	10	cctccacacaggcttctctgactt	cctaacttgcttgagttattgccc	134–158
CSRD247	14	ggacttgccagaactctgcaat	cactgtggtttgtattagtcagg	220–247
McM527	5	gtccattgcctcaaatcaattc	aaaccacttgactactccccaa	165–187
ILSTS087	28	agcagacatgatgactcagc	ctgcctcttttcttgagag	135–155
BM6444	2	ctctgggtacaacactgagtcc	tagagagtttccctgtccatcc	118–200
P19 (DYA)	20	aacaccatcaaacagtaagag	catagtaacagatcttcctaca	160–196
TCRVB6	Unknown	gagtcctcagcaagcaggtc	ccaggaattggatcacacct	217–255
DRBP1	23	atggtgcagcagcaaggtgagca	gggactcagtctctctatctctttg	195–229
ETH10	5	gttcaggactggccctgctaaca	cctccagcccactttctcttctc	200–210

**Table 2:** Microsatellite markers and observed number of alleles.

Loci	Population								
	Kilis	Yayladag	Shami	Honamli	Saanen	Hair	Angora	Alpine	*Total*
SPS113	7	6	6	7	9	7	10	5	11
McM527	8	5	6	7	6	7	6	6	10
CSRD247	10	7	9	8	6	8	9	8	11
BM6444	16	13	18	17	15	20	25	10	33
ILSTS087	7	5	6	8	7	9	8	6	10
TCRVB6	12	10	9	11	9	11	12	8	14
DRBP1	6	8	6	6	4	6	8	4	14
MAF70	7	11	11	9	9	11	9	7	18
ETH10	4	6	5	5	4	6	8	4	14
P19 (DYA)	8	7	11	10	10	9	8	7	13
INRA023	8	8	9	6	4	6	8	5	12

Mean	8.45	7.82	8.73	8.55	7.55	9.09	10.09	6.36	**14.55**

**Table 3:** Observed (Ho) and expected (He) heterozygosities at eleven microsatellite loci.

Loci	Ho/He	Population							
		Kilis (*n* = 32)	Yayladag (*n* = 32)	Shami (*n* = 32)	Honamli (*n* = 32)	Saanen (*n* = 28)	Hair (*n* = 32)	Angora (*n* = 43)	Alpine (*n* = 13)
SPS113	Ho	0.600	0.692	0.781	0.767	0.893	0.862	0.707	0.923
	He	0.729	0.661	0.709	0.756	0.821	0.807	0.801	0.769

McM527	Ho	0.700	0.704	0.594	0.700	0.704	0.759	0.675	0.692
	He	0.761	0.604	0.733	0.704	0.645	0.754	0.690	0.710

CSRD247	Ho	0.833	0.926	0.844	0.967	0.607	0.767	0.805	0.846
	He	0.819	0.791	0.797	0.845	0.612	0.812	0.842	0.793

BM6444	Ho	0.875	0.875	0.938	0.903	0.704	0.774	0.805	0.833
	He	0.863	0.863	0.917	0.877	0.867	0.851	0.903	0.858

ILSTS087	Ho	0.469	0.656	0.656	0.813	0.704	0.781	0.575	0.727
	He	0.484	0.635	0.667	0.756	0.769	0.748	0.525	0.702

TCRVB6	Ho	0.875	0.969	0.781	0.844	0.630	0.871	0.744	0.692
	He	0.896	0.854	0.853	0.819	0.755	0.869	0.858	0.669

DRBP1	Ho	0.281	0.406	0.387	0.188	0.464	0.219	0.550	0.222
	He	0.738	0.669	0.687	0.446	0.576	0.487	0.747	0.451

MAF70	Ho	0.677	0.688	0.806	0.742	0.643	0.813	0.732	0.769
	He	0.779	0.763	0.836	0.797	0.738	0.796	0.784	0.757

ETH10	Ho	0.531	0.281	0.484	0.688	0.321	0.625	0.800	0.750
	He	0.703	0.579	0.673	0.558	0.427	0.608	0.704	0.681

P19 (DYA)	Ho	0.625	0.452	0.833	0.719	0.643	0.656	0.860	0.700
	He	0.767	0.789	0.821	0.821	0.841	0.815	0.855	0.810

INRA023	Ho	0.688	0.935	0.900	0.625	0.519	0.548	0.667	0.500
	He	0.691	0.817	0.835	0.488	0.445	0.445	0.733	0.705

Mean	Ho	**0.650**	**0.689**	**0.728**	**0.723**	**0.621**	**0.698**	**0.720**	**0.696**
	He	**0.748**	**0.729**	**0.775**	**0.715**	**0.681**	**0.727**	**0.767**	**0.719**

**Table 4:** *F*_ST_ (lower diagonal) and *D*_*A*_ (upper diagonal) values.

	Kilis	Yayladag	Shami	Honamli	Saanen	Hair	Angora	Alpine
Kilis	—	0.252	0.131	0.191	0.225	0.176	0.176	0.221
Yayladag	0.075^*∗∗∗*^	—	0.254	0.215	0.321	0.207	0.285	0.293
Shami	0.038^*∗∗∗*^	0.072^*∗∗∗*^	—	0.178	0.226	0.170	0.207	0.248
Honamli	0.079^*∗∗∗*^	0.080^*∗∗∗*^	0.072^*∗∗∗*^	—	0.194	0.071	0.247	0.269
Saanen	0.110^*∗∗∗*^	0.125^*∗∗∗*^	0.104^*∗∗∗*^	0.070^*∗∗∗*^	—	0.174	0.262	0.262
Hair	0.074^*∗∗∗*^	0.078^*∗∗∗*^	0.075^*∗∗∗*^	0.005 ns	0.062^*∗∗∗*^	—	0.210	0.254
Angora	0.049^*∗∗∗*^	0.086^*∗∗∗*^	0.057^*∗∗∗*^	0.090^*∗∗∗*^	0.108^*∗∗∗*^	0.078^*∗∗∗*^	—	0.198
Alpine	0.054^*∗∗∗*^	0.099^*∗∗∗*^	0.071^*∗∗∗*^	0.094^*∗∗∗*^	0.090^*∗∗∗*^	0.084^*∗∗∗*^	0.046^*∗∗∗*^	—

(^*∗∗∗*^ *P* < 0.001, ns: nonsignificant.)
